# Involvement of dopamine D_2_ ‐like receptors in the antiepileptogenic effects of deep brain stimulation during kindling in rats

**DOI:** 10.1111/cns.14059

**Published:** 2022-12-13

**Authors:** Mahmoud Rezaei, Samireh Ghafouri, Azam Asgari, Victoria Barkley, Yaghoub Fathollahi, Sareh Rostami, Amir Shojaei, Javad Mirnajafi‐Zadeh

**Affiliations:** ^1^ Department of Physiology, Faculty of Medical Sciences Tarbiat Modares University Tehran Iran; ^2^ Department of Biology University of Victoria Victoria British Columbia Canada; ^3^ Krembil Research Institute University Health Network Toronto Ontario Canada; ^4^ Institute for Brain Sciences and Cognition Tarbiat Modares University Tehran Iran

**Keywords:** deep brain stimulation, dentate gyrus, dopamine D_2_‐like receptors, kindling, sulpiride

## Abstract

**Aims:**

Deep brain electrical stimulation (DBS), as a potential therapy for drug resistive epileptic patients, has inhibitory action on epileptogenesis. In the present investigation, the role of dopamine D_2_‐like receptors in the antiepileptogenic action of DBS was studied.

**Methods:**

Seizures were induced in adult rats by stimulating the perforant path in a semi‐rapid kindling method. Five minutes after the last kindling stimulation, daily DBS was applied to the perforant path at the pattern of low frequency stimulation (LFS; 1 Hz; pulse duration: 0.1 ms; intensity: 50–150 μA; 4 trains of 200 pulses at 5 min intervals). Sulpiride (10 μg/1 μl, i.c.v.), a selective dopamine D_2_‐like receptor antagonist, was administered prior to the daily LFS application.

**Results:**

Kindling stimulations increased cumulative daily behavioral seizure stages, daily afterdischarge duration (dADD), and population spike amplitude (PS) in dentate gyrus following perforant path stimulation, while applying LFS decreased the kindled seizures' parameters. In addition, kindling potentiated the early (at 10–50 ms inter‐pulse interval) and late (at 150–1000 ms inter‐pulse interval) paired‐pulse inhibition and decreased the paired‐pulse facilitation (at 70–100 ms inter‐pulse interval). These effects were also inhibited by applying LFS. All inhibitory effects of LFS on kindling procedure were prevented by sulpiride administration.

**Conclusion:**

These data may suggest that LFS exerts its preventive effect on kindling development, at least partly, through the receptors on which sulpiride acts which are mainly dopamine D_2_‐like (including D_2_, D_3_, and D_4_) receptors.

## Graphical abstract

Applying low‐frequency stimulation reduces kindling rate and prevents the kindling‐induced changes in field potential recordings. These effects are somehow through the receptors on which sulpiride acts which are mainly dopamine D_2_‐like (including D_2_, D_3,_ and _D4_) receptors.

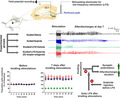

## 1 INTRODUCTION

In spite of introducing a growing number of antiepileptic drugs, almost 20%–30% of epilepsy patients are resistant to medical treatment, and many of them cannot be operated due to challenges when identifying seizure foci.^1^ Therefore, new therapeutic strategies for these patients are necessary. Low‐frequency electrical stimulation (LFS), as a common pattern in inducing long‐term depression (LTD) and depotentiation, has a long‐lasting inhibitory effect on epileptic seizures.^2^

Our previous studies which examined the effect of perforant path LFS on rapid perforant path kindled rats using evoked field potential recording showed that LFS delayed the development of kindled seizures, so that the number of stimulations to achieved different kindled seizure stages increased when applied following each kindling stimulation. In addition, LFS prevented the kindling‐induced synaptic potentiation and inhibited the increase in paired‐pulse depression.^3^, ^4^ However, LFS’ precise antiepileptogenic mechanism is unknown. The involvement of neurotransmitters and/or neuromodulators, including adenosine,^5^ serotonin,^6^ endocannabinoids^7^ and galanin^4^ in LFS' antiepileptogenic effects has been reported previously.

Dopamine, as an important neuromodulator, has a crucial role in synaptic plasticity,^8^ neural excitability and seizure modulation.^9^, ^10^ Dopamine exerts its effects through two kind of receptors, including D_1_‐like (i.e., D_1_ and D_5_) and D_2_‐like (i.e., D_2_, D_3,_ and D_4_) receptors.^11^ Dopamine plays a major role in controlling seizures arising from the limbic system. The effects of D_1_‐like and D_2_‐like receptors on seizure activity are opposite to each other. D_1_‐like receptors have a pro‐epileptogenic effect through activating the Gs protein signaling pathway. In contrast, D_2_‐like receptors exert an antiepileptogenic effect by activating the Gi protein signal transduction.^10^ Our previous experiments provided evidence regarding the probable involvement of the Gi protein signaling pathway in exerting the LFS' antiepileptogenic effects.^12^

There are conflicting reports about the impact of various dopamine receptors in synaptic plasticity. Most studies have demonstrated that dopamine D_1_/D_5_ receptors are involved in the long‐term potentiation (LTP) induction^13^, ^14^, ^15^ and D_2_‐like receptor activation increases LTD induction.^16^ Recently, we showed that applying LFS in the hippocampus exerted its antiepileptic effects on fully kindled mice by reducing the amplitude and slope rise of excitatory and inhibitory postsynaptic currents in CA1 cells as well as restoring kindled‐induced impairment in learning and spatial memory. These effects were blocked by dopamine D_2_‐like receptor antagonist and were mimicked by dopamine D_2_‐like receptor agonist.^17^

Considering the similarities between kindling‐induced synaptic potentiation and LTP^18^ and regarding the important role of dopamine in synaptic plasticity, as well as the antiepileptic and antiepileptogenic effects of dopamine D_2_‐like receptors, the aim of this study was to investigate the involvement of dopamine D_2_‐like receptors in the role of LFS on the development of epileptogenesis during a perforant path kindling procedure.

## 2 METHODS

### 2.1 Animals

Forty‐two adult male Wistar rats (270–290 g; Pasteur Institute of Iran, Tehran) were used. Animals were kept at in the animal facility with an ambient temperature of 25 ± 2°C, under 12:12 light/dark program. After surgery, rats were kept in individual plastic cages with woodchip bedding and allowed free access to standard food and water. Efforts were made to reduce the animal's suffering and the number of animals used. All experimental procedures were done according to the guidelines of “Ethical Committee of School of Medical Sciences, Tarbiat Modares University” that was in accordance with the “National Institutes of Health guide for the care and use of Laboratory animals.”

### 2.2 Animal Surgery

Animal surgery was conducted as mentioned previously.^3^, ^19^ Rats were anesthetized by a mixture of ketamine (100 mg/kg) and Xylazine (10 mg/kg) (Alfasan) and an anesthesia booster was injected after 45–50 min. A stereotaxic instrument (Stoelting, Wood Dale) was used for electrode and cannula implantation while the incisor bar was adjusted 3.3 mm below interaural line. A bipolar stimulating electrode (two twisted electrodes with a tip distance of 0.5 mm) was implanted in the perforant path (coordinates: AP: −6.9 mm; ML: 4.1 mm; and, DV: 2–2.5 mm below dura) and a monopolar recording electrode implanted in the dentate gyrus (coordinates: AP: −2.8 mm; ML: 1.8 mm; and, DV: 2.5–3 mm below dura) of the right hemisphere (according to the atlas of Paxinos and Watson (2007)^20^). The electrodes' depth was adjusted based on electrophysiological indices, so that a large population spike (PS) amplitude could be recorded in the dentate gyrus in response to the perforant path stimulation. Electrodes (A‐M Systems) were stainless steel, Teflon coated, 127 μm in diameter, and insulated except at their tips.

To achieve icv (intracerebroventricular) injection of chemicals, a guide cannula (23‐gauge) was inserted in the right lateral ventricle (coordinates: AP: −0.8 mm; ML: −1.4 mm and 2.6 mm below dura). The reference and ground electrodes were attached to stainless steel screws and were located in the skull above the frontal and occipital cortices of left hemisphere. Small metal pins were connected to the end of all electrodes, and through them, the electrodes were attached to a multichannel socket (one pair of 2‐pin relimate connector female). The socket was stable on the skull by dental acrylic cement.

The recovery period was 10 days. Then, the electrophysiological signals were recorded when the animal was moved freely in the recording box inside the Faraday cage. Evoked field potential recording was done when the animal had no motion and awake.

### 2.3 Semi‐rapid kindling procedures

Semi‐rapid kindling was done, as described previously.^3^, ^4^ To achieve the afterdischarge (AD) threshold, 3 s train of monophasic square waves (1 ms pulse duration) was applied at 50 Hz (BIODAC ES1721, TRITA Health Technology CO.). The stimulation intensity was started from 30 μA and increased in steps of 10 μA at 10 min intervals. The minimum intensity that could generate Ads for at least 10 s was considered the AD threshold and used for stimulation. AD threshold intensity was from 50–150 μA. Each rat was stimulated 12 times/day at 10 min intervals. Following stimulation, Ads’ duration and the behavioral progression of kindling (seizure stages 1–5; according to Racine scores^21^) were measured.

### 2.4 Evoked field potential recording

**FIGURE 1:**
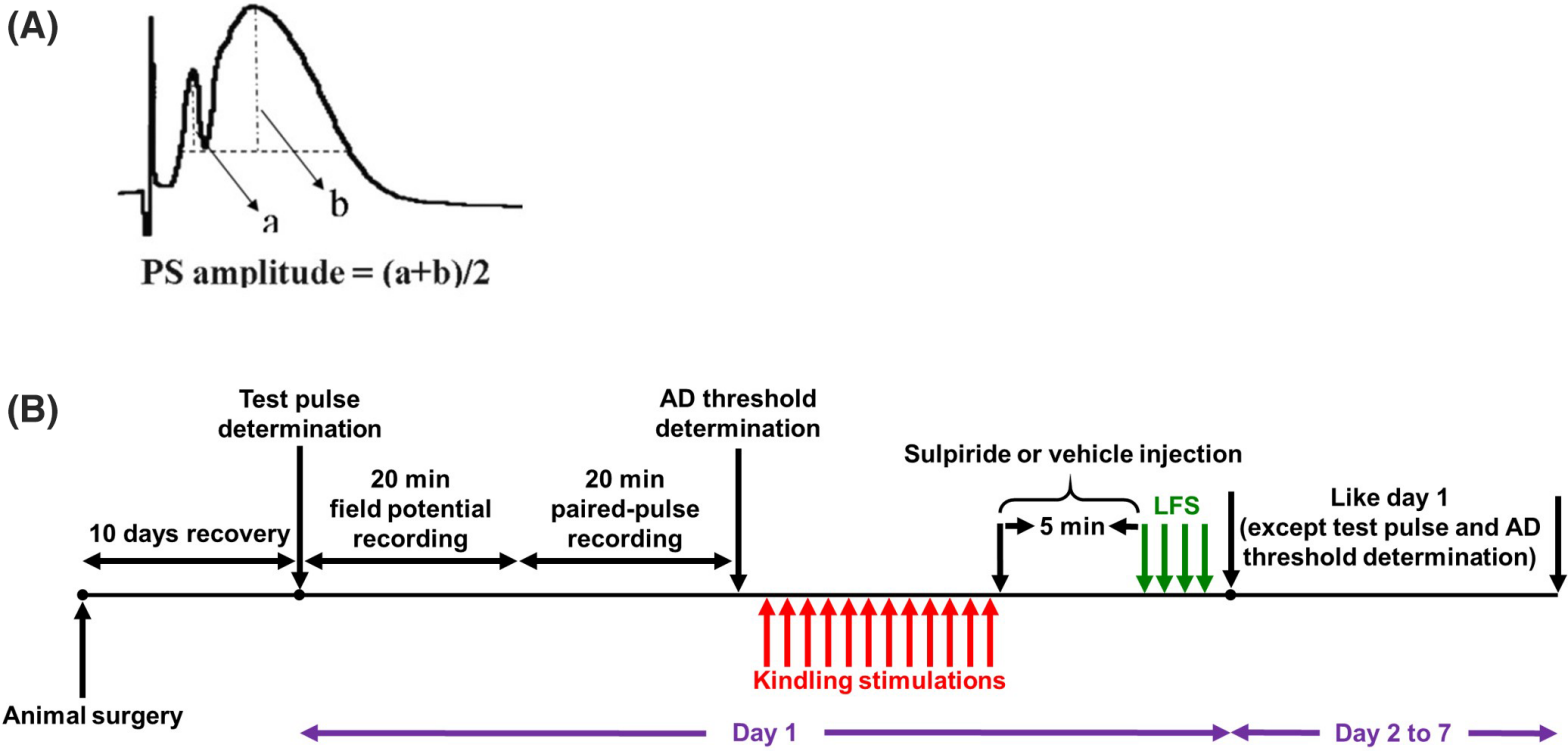
(A) PS amplitude was calculated by averaging the height from the peak of the pEPSP to maximum downward deflection of PS by averaging lines a and b. (B) Timeline diagram showing the experimental protocol used in kindled and KLFS groups. Red and green arrows indicate kindling and LFS stimulation, respectively.

Evoked field potentials were recording while the perforant path was stimulated at test pulse intensity and evoked field potentials were recorded from the dentate gyrus. The test pulse intensity was determined as the intensity that induced 50% of maximum population spike (PS) amplitude. To obtain the test pulse intensity, input/output curve was achieved by applying single 0.1 ms monophasic square wave pulses to the perforant path at different intensities from 100 μA up to 800 μA. There was a 10 s interval between consecutive stimulations, and the evoked field potentials were recorded in the dentate gyrus. Then, PS amplitude was calculated as the average height from the peak of the population excitatory postsynaptic potential (pEPSP) to the base of PS, as shown in Figure 1A. The test pulse amount for different animals was from 250 to 700 μA. pEPSP slope and PS amplitude were measured on the days 1, 4, and 7 of the kindling procedure.

All responses were amplified 500 times, filtered (1–3000 Hz band pass filters), and digitized at the sampling rate of 10 kHz, using a data acquisition system (BIODAC ES1721, TRITA Health Technology CO.). Digital data were transferred to the computer, and data were analyzed using custom MATLAB script.

Paired‐pulse stimulations were also run at the intensity of test pulse and inter‐pulse intervals of 20, 30, 40, 70, 100, 300, 500, and 1000 ms. These intervals were used randomly at 0.1 Hz, and six sweeps were averaged at each inter‐pulse interval. The paired‐pulse index was defined as the percent ratio of the second PS to the first PS. Paired‐pulse indices were calculated on 1st, 4th and 7th day before applying the kindling stimulations.

### 2.5 Drugs

(S)‐5‐Aminosulfonyl‐N‐[(1‐ethyl‐2‐pyrrolidinyl)methyl]‐2‐methoxybenzamide (Sulpiride; Sigma‐Aldrich), a selective dopamine D_2_‐like receptor antagonist, was dissolved in a drop of glacial acetic acid and its pH adjusted to 6 with addition of 1 N NaOH^22^ in a manner that drug was 10 micrograms per microliter of solution. The solution was then sterilized using a microfilters (0.2 μm, Schleicher & Schuell). Using a microsyringe pump (Stoelting), drugs were administered via a 30‐gauge cannula into the lateral ventricle at the volume of 1 μl over 2 min. The drug solution was injected daily, 5 min before LFS application.

### 2.6 Experimental design

Animals were divided into four groups. In kindled groups, animals received kindling stimulation with a semi‐rapid kindling procedure, and the kindling parameters (seizure stages and daily Afterdischarge duration (dADD)) were measured. Field potential parameters (PS amplitude and pEPSP slope) were measured every day before kindling stimulations for 20 min, and data were used as baseline. As mentioned above, paired pulse indices were also defined on days one, four and seven after field potential recording. In the kindled + LFS (KLFS) groups, LFS (0.1 ms pulse duration at 1 Hz, 4 trains of 200 pulses at 5 min intervals at the intensity of AD threshold) was applied daily at 5 minutes after the last kindling stimulation. LFS parameters were determined based to our preliminary experiments.^3^, ^23^ In the LFS group, only LFS (without kindling stimulations) were applied. In control group, animals received no stimulation. Both kindled and KLFS groups were then divided into three subgroups in which animals received (a) sulpiride before each LFS stimulations (kindled + Sulpiride and KLFS + Sulpiride), (b) vehicle before each LFS stimulation (kindled + Vehicle and KLFS + Vehicle) and (c) no injection (kindled and KLFS). The timeline of experimental protocol has been shown in Figure 1B.

The kindling stimulation days were continued until the animals showed at least one, stage 5 seizure. The day that a given animal showed a stage 5 seizure was accepted as the last experimental day for that animal. The mean number of stimulation days to reach a stage 5 seizure in kindled and kindled + Vehicle groups was 6.8 ± 0.5 and 7.1 ± 0.3, respectively (there was no statistically significant difference between the daily seizure stage and daily ADD). Therefore, the 7th day was considered as the last experimental day in other experimental groups. In addition, field potential parameters were also analyzed during the first, fourth, and seventh days of experiments in different groups. At least five rats were used in each group.

### 2.7 Statistical analysis

Data were shown as mean ± standard error of mean (S.E.M.) and by the number of observations. The data averaging was done for 12 successive evoked responses to evaluate the synaptic responses. In the case of paired‐pulse and input/output curve experiments, six successive evoked responses were averaged. PS amplitude and pEPSP slope were presented as the percent of baseline and daily cumulative AD duration (cADD) was measured as the sum of AD durations recorded during 12 daily stimulations. A Kolmogorov‐Smirnov test was used to assess data distribution. A two‐way repeated measures ANOVA was used to determine the changes in different parameters. Statistically significant differences were analyzed further by a Tukey's post hoc test. For analyzing the effect of LFS on cumulative behavioral seizure scores, the nonparametric Kruskal‐Wallis and Mann‐Whitney U‐tests were used. Data from two independent groups were compared by using a Student unpaired *t*‐test. The probability level (*p* value) of less than 0.05 was considered statistically significant.

## 3 RESULTS

No significant difference observed in test pulse intensity among experimental groups on the first day of stimulation (Table 1). There was no difference in synaptic strength and excitability between animals at the beginning of the experiments (Please see Figure 3E). Meanwhile, microinjection of the vehicle (glacial acid acetic + NaOH) did not produce any significant difference in measured parameters. Thus, the parameters of animals receiving sulpiride were compared with their respective vehicle‐receiving groups.

**TABLE 1:** The magnitude of afterdischarge (AD) threshold and test pulse in different experimental groups.

Experimental group	AD threshold (μA)	Test pulse (μA)
Kindled	80.1 ± 6.5	392.6 ± 43.1
Kindled + sulpiride	76.0 ± 11.2	341.4 ± 31.5
KLFS	114.3 ± 18.0	429.9 ± 40.7
KLFS + sulpiride	106.0 ± 25.2	367.8 ± 65.1
Control	–	267.3 ± 31.1
LFS	–	397.3 ± 34.7

*Note*: Values are mean ± SEM. There was no significant difference between none of parameters in different experimental groups.

### 3.1 Effect of dopamine D_2_ ‐like receptor blockade on LFS' antiepileptogenic action

**FIGURE 2:**
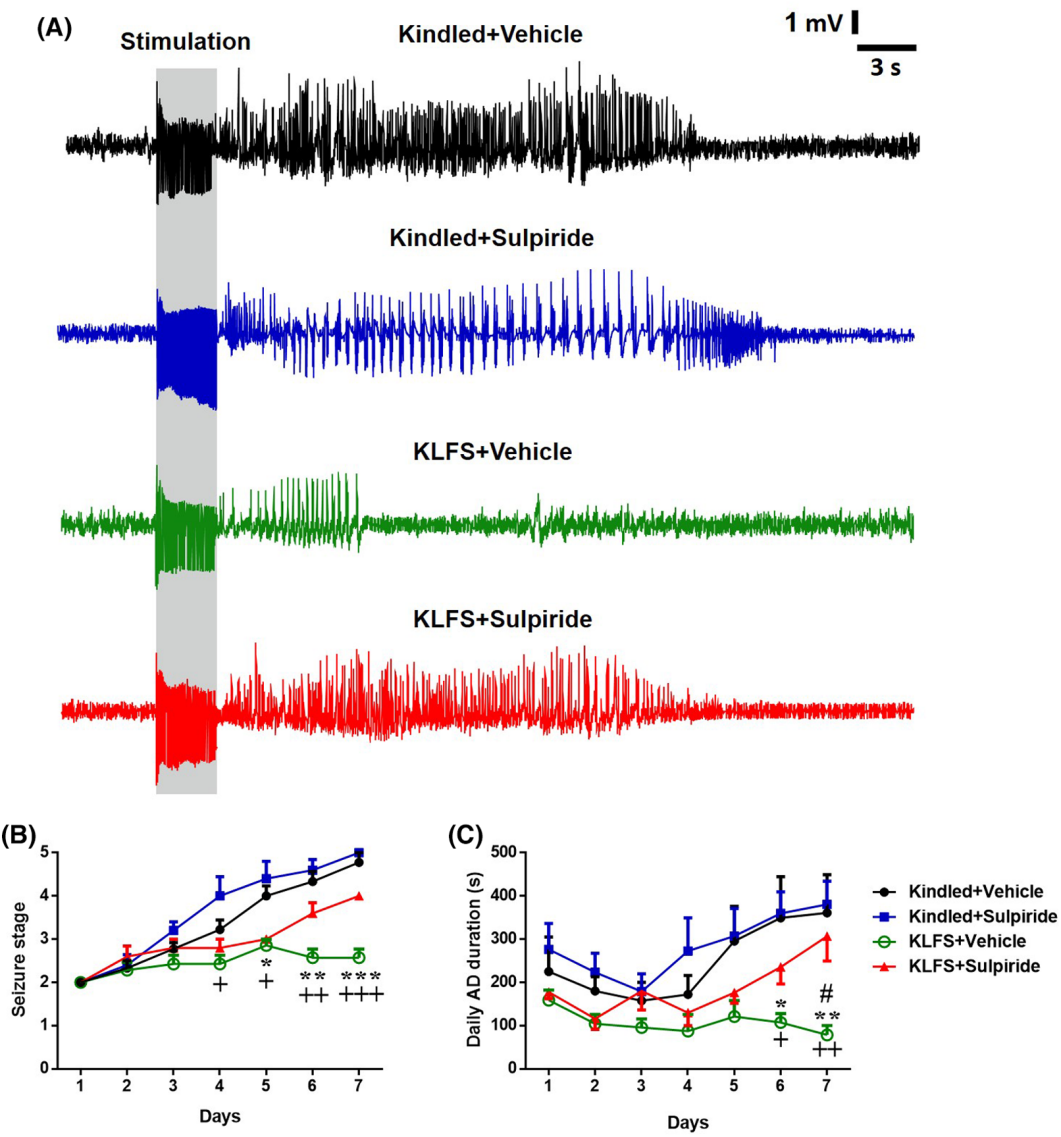
Effect of sulpiride microinjection (10 μg/1 μl) on the inhibitory action of LFS on seizure stages and afterdischarges (ADs). (A) Sample records of ADs in different experimental groups after the first kindling stimulation on the 7th day, (B) changes in seizure stages, and (C) daily AD duration (ADD) during 7 days of kindling development. There was a significant decrease in seizure stages from the 5th day to 7th days of kindling procedure in KLFS group. Daily ADD in the 6th and 7th day in Kindled + vehicle and KLFS + vehicle groups showed a significant difference. Values are mean ± S.E.M. (*n =* 6). *p < 0.05, **p < 0.01 and ****p < 0.001 when compared with the Kindled + vehicle group; #*p <* 0.05 when compared with the KLFS + Sulpiride group.

The magnitude of AD threshold among experimental groups showed no significant difference on the first day of experiments (Table 1). Therefore, the seizure susceptibility was similar among all different experimental groups at the beginning of the experiments. Preliminary experiments showed that microinjection of sulpiride at the dose of 10 (but not 20) μg/1 μl during the first 7 days of kindling acquisition had no significant effect on seizure parameters and daily ADD in kindled + Sulpiride compared to kindled + Vehicle group (Figure 2). Thus, the dose of 10 μg/1 μl was injected in KLFS + Sulpiride group to test the role of dopamine D_2_‐like receptors in the antiepileptogenic effect of LFS. These data were in line with a previous report which showed sulpiride alone had no effect on different seizure models.^24^ As reported previously,^3^ LFS had inhibitory effect on kindling acquisition and decreased the behavioral seizure stages. LFS‐receiving animals in the KLFS + Vehicle group did not show stages 4 and 5 seizures after 7 days and the increase in the duration of their ADs was less than kindled + Vehicle group (Figure 2A). The seizure stages of animals in KLFS + Vehicle group was less than kindled + Vehicle group during days 5–7 of stimulations (Figure 2A, B). Sulpiride administration decreased the inhibitory effect of LFS on the kindling rate in KLFS + Sulpiride group significantly, so that seizure stages increased in KLFS + Sulpiride compared to the KLFS + Vehicle group. In addition, daily ADD was significantly increased in KLFS + Sulpiride compared to KLFS + Vehicle in the sixth and seventh days of stimulation. On the other words, there was no significant difference between KLFS + Sulpiride and kindled + Vehicle groups (Figure 2C).

### 3.2 Effect of Dopamine D_2_ ‐like receptors blockade on LFS' inhibitory action on field potential recordings

**FIGURE 3:**
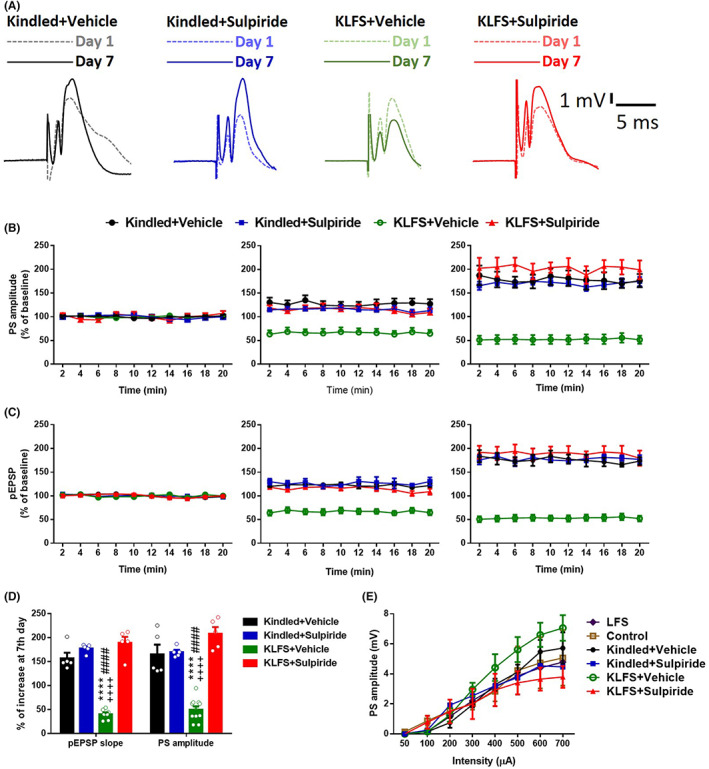
Effect of sulpiride microinjection (10 μg/1 μl) on the inhibitory action of LFS on kindling‐induced potentiation in PS amplitude and pEPSP slope. (A) Sample averages of 12 waveforms taken on days 1 and 7 of kindling procedure. Time‐course diagrams showing the changes in PS amplitude (B) and pEPSP slope (C) in the Kindled + vehicle (*n* = 7), KLFS + vehicle (*n* = 7), and KLFS + Sulpiride (*n* = 5) groups during the first 7 days of kindling acquisition. Each graph shows 20 min baseline perforant path‐evoked responses recorded before kindling stimulation on days 1,4 and 7. (D) Percent of changes at the 7th day in PS amplitude and pEPSP responses. Values are mean ± S.E.M. ****p < 0.001 when compared with the KLFS + vehicle group.

The basal synaptic transmission was potentiated following the kindling stimulations. In the kindled + Vehicle group, there was 70.6% ± 3.11% increment in pEPSP slope and 73.3% ± 13.3% increase in PS amplitude after 7 days of the kindling procedure. (Figure 3A–C). Applying LFS suppressed the kindling‐induced potentiation significantly.

In the KLFS + Vehicle group, the increased percentage of pEPSP slope and PS amplitude were 47.8% ± 2.4% and 50.2% ± 6.9%, respectively, during 7 days of the kindling procedure (Figure 3B,C). A sulpiride microinjection (10 μg/1 μl) in kindled + Sulpiride group had no significant effect on evoked field potential parameters (Figure 3). This was in line with a previous report that showed sulpiride alone had no significant effect on synaptic transmission in brain slices.^25^ However, sulpiride (10 μg/1 μl) significantly decreased the inhibitory effect of LFS on field potential parameters. There was 98.4% ± 1.5% increase in the pEPSP slope and 99.4% ± 12.6% increase in PS amplitude in the KLFS + Sulpiride group following 7 days of kindling stimulations (compared to the respective data on the first day). Therefore, no significant difference was observed between KLFS + Sulpiride and kindled + Vehicle groups (Figure 3D).

### 3.3 Effect of Dopamine D_2_ ‐like receptors blockade on LFS' suppressing effect on paired pulse measurements

**FIGURE 4:**
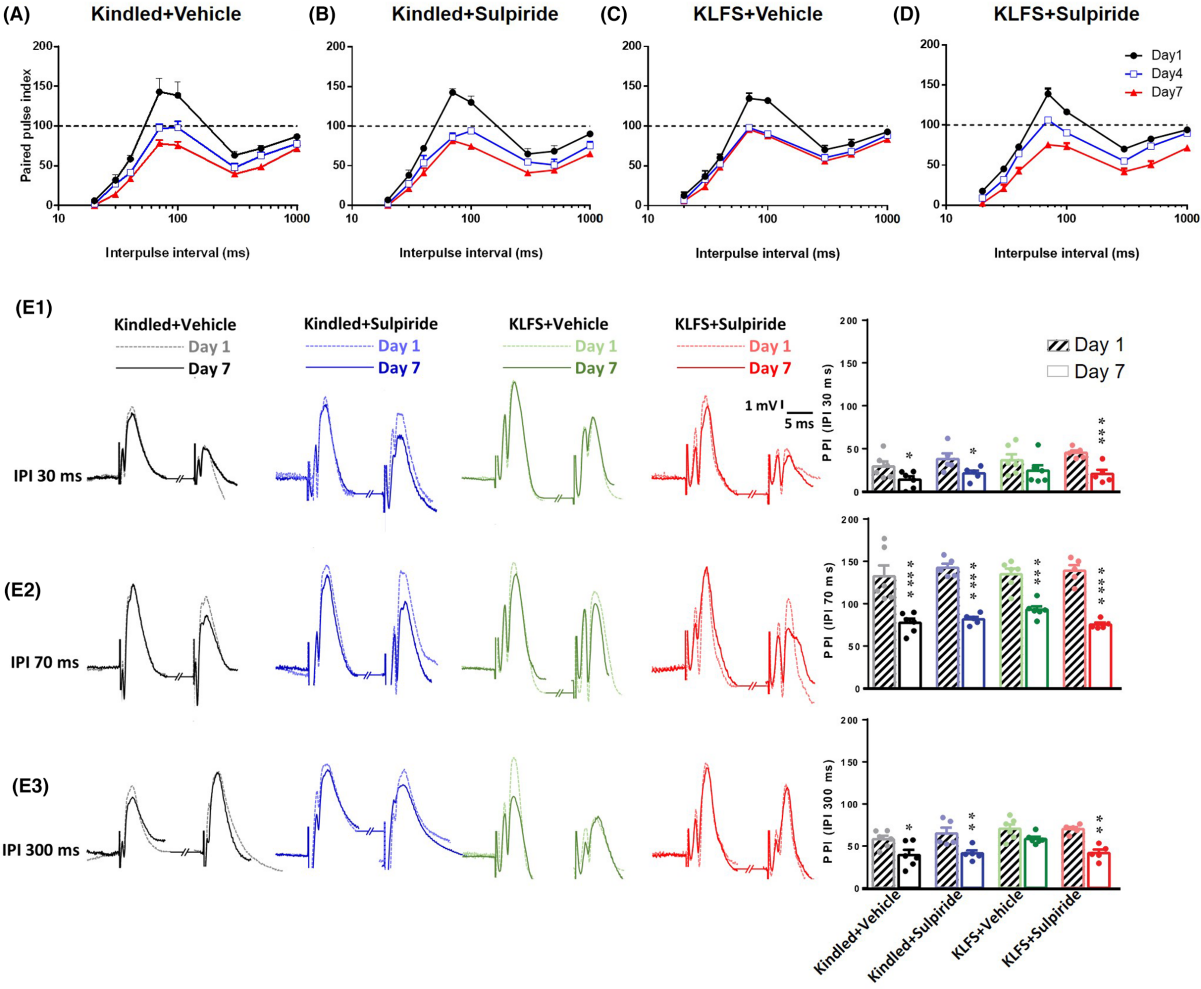
Paired‐pulse indices in Kindled + vehicle (A), Kindled + Sulpiride (B), KLFS + vehicle (C), and KLFS + Sulpiride (D) group. (E1) Sample records showing early paired‐pulse depression at inter‐pulse interval of 30 ms in different experimental groups. The column bar graph on the right shows the measured paired‐pulse index on day 1 and day 7 in experimental groups. (E2) The same parameters for paired‐pulse facilitation at 70 ms inter‐pulse interval and (E3) show the same parameters for late paired‐pulse depression at 300 ms inter‐pulse interval. Values are mean ± S.E.M. *p < 0.05, **p < 0.01, ***p < 0.001, and ****p < 0.0001 when compared with the data on day 1 of the respected group.

Paired pulse indices were calculated based on the measured PS amplitudes in response to the paired stimuli. As reported previously,^3^, ^26^, ^27^, ^28^ early and late paired pulse depression increased and paired pulse facilitation decreased during kindling procedure (Figure 4A). LFS prevented all of these changes when applied in the KLFS + vehicle group (Figure 4B). This effect of LFS was reduced following microinjection of sulpiride (10 μg/1 μl) in the KLFS + Sulpiride group (Figure 4C). Sulpiride microinjection had no significant effect on kindling‐induced changes in paired‐pulse indices (Figure 4).

For better data presentation, we selected the inter‐pulse intervals of 30, 70 and 300 ms in which early paired‐pulse depression, paired‐pulse facilitation and late paired‐pulse depression occurred. Then, the changes in the paired‐pulse index at a given paired‐pulse interval were compared between the seventh and the first day of experiment. In the kindled + Vehicle group, the paired‐pulse index was reduced at 30, 70 and 300 ms intervals on the seventh day significantly. The decrement of paired‐pulse index at inter‐pulse intervals of 30 and 300 ms was considered as potentiation of paired pulse depression. However, the decrement of the paired‐pulse index at inter‐pulse interval of 70 ms demonstrated a reduction in paired pulse facilitation. The observed changes in paired‐pulse index at paired‐pulse intervals of 30 and 300 ms (but not at 70 ms) reduced when LFS was applied after kindling stimulations. However, in the presence of sulpiride, LFS could not exert its restoring action, showing the role of D_2_‐like receptors in inhibitory action of LFS. Sulpiride had no significant effect on changes in paired‐pulse index on the seventh day in kindled + Sulpiride group compared to the first day (Figure 4).

## 4 CONCLUSION

Our findings demonstrated that inhibitory effects of LFS on perforant kindling progression, including its postponement of the expression of behavioral seizure stages and its preventative action on potentiation of synaptic responses following kindling stimulations, are partly related to dopamine D_2_‐like receptors activity.

Most previous studies have shown the antiepileptic role of dopamine D_2_‐like receptors.^29^, ^30^, ^31^ For instance, LY171555 (a dopamine D_2_ receptor agonist) significantly decreased the kindling parameters (such as seizure intensity, seizure duration and AD duration) in fully kindled animals when this agonist is microinjected into the nucleus accumbens at 15 min before kindling.^10^ In addition, clinical and experimental studies reported the neuromodulatory role of dopamine in epilepsy,^32^, ^33^, ^34^, ^35^ and dopamine is considered an important regulator for seizure activity.^36^, ^37^ In animal models of temporal lobe epilepsy, dopamine level and dopaminergic neuronal activity increases.^38^ Our results are consistent with this action of dopamine D_2_‐like receptors because applying sulpiride (as a D_2_‐like receptor antagonist) at a dose that had no significant effect on semi‐rapid kindling parameters, prevented the antiepileptogenic effects of LFS. Accordingly, the sulpiride's preventative effects on LFS' inhibitory action (in KLFS + Sulpiride group) may be related to the role of D_2_‐like receptors in mediating LFS' action, but not to antiepileptogenic effects of sulpiride.

It has to be mentioned that using a conventional kindling protocol (one stimulation every day) seems to be more favorable, relative to the semi‐rapid kindling protocol, to examine the effects of sulpiride in mediating the anti‐epileptogenic effect of LFS. However, because we tried to record evoked field potentials in freely moving animals, it was very difficult to record field potentials in animals for a long duration in conventional kindling protocol.

In the present study, sulpiride was microinjected intracerebroventricularly. While administration of sulpiride via systemic injection could provide stronger clinical relevance. Most of researchers believe that sulpiride penetrates the blood‐brain barrier poorly because of its low lipid solubility. Interestingly, sulpiride is the substrate of a class of organic cation transporters. These transporters may increase the penetration of sulpiride through the blood‐brain barrier the increase its distribution in the brain.^39^

The precise antiepileptogenic and/or anticonvulsant effects of LFS have not been completely determined. In this regard, it has been suggested that mechanisms similar to long‐term depression (LTD) or depotentiation may be involved. Our recent study suggested the involvement of a “depotentiation‐like effect” in anticonvulsant action of LFS.^17^ However, it is not clear whether the same mechanism involves in antiepileptogenic effect of LFS. However, similar to previous studies,^2^, ^40^, ^41^ the present data also showed that applying LFS immediately after kindling stimulation prevented the kindling‐induced potentiation of synaptic transmission and retarded epileptogenesis. Therefore, it may be posited that preventing the abnormal potentiation in synaptic transmission possibly is involved in antiepileptogenic effects of LFS. For a better evaluation of the antiepileptogenesis effect of LFS action and assessment of sulpiride effects, it would be useful to measure the changes in local field potentials post‐LFS applying (shortly following each LFS or after four LFS trains). In this way, analyzing the local field potentials and brain waves could provide valuable information about LFS action and this suggestion has to be considered in the future studies. In addition, Assessment of evoked potentials and paired responses shortly after LFS, in the presence or absence of sulpiride, may provide useful information.

Dopamine has strong modulatory role on synaptic potentiation. However, there are many controversies about the effect of D_2_‐like receptors on synaptic potentiation. Some studies showed that D_2_‐like receptor agonists inhibit depotentiation in dentate gyrus^16^ and inhibit LTD in the hippocampal CA1 region.^42^ However, other studies showed LTP enhancement following the application of a D_2_‐like receptor antagonist. This LTP augmentation was also observed in mice lacking D_2_‐like receptors.^43^, ^44^ In another study, the genetic deletion of D_2_ receptors, and using D_2_‐like receptor antagonists, severely impaired LTD in the CA1.^44^ It was also reported that depotentiation is generated through the activation of D_4,_ but not D_1_/D_5,_ receptors in the hippocampus.^45^ Therefore, it may be suggested that the inhibitory action of sulpiride on antiepileptogenic effect of LFS may show D_2_‐like receptors' involvement in intervening the LFS' action in producing LTD and/or depotentiation following its application during the kindling procedure.

The firing pattern of dopaminergic fibers may be phasic (10–30 Hz) or tonic (1–4 Hz).^46^, ^47^ Considering the high‐affinity of D_2_‐like receptors, tonic firing of dopaminergic fibers at low‐frequencies, which is responsible for the basal level of dopamine, mainly activated these receptors. On the other hand, dopamine levels rise transiently during phasic firing pattern of dopaminergic fibers, and in this situation the low‐affinity D_1_‐like receptors may be activated. Accordingly, laboratory animal models of seizures (e.g., kindling) are accompanied with increased firing of dopaminergic neurons and rising dopamine release and concentration.^38^, ^48^ In this situation, D_1_‐like receptors that have a pro‐epileptogenic effect may activate. In this study, the LFS frequency (1 Hz) was near the tonic firing pattern and through connectivity between hippocampus and ventral tegmental area (as the main hippocampal dopamine source) might induce tonic pattern and decrease the amount of dopamine. For that reason, at this range of dopaminergic neurons activity, the situation was suitable for D_2_‐like receptors activity and their antiepileptogenic effects.

It may also be suggested that following sulpiride administration, the released dopamine could activate only D_1_‐like receptors. Activation of these receptors, which couple to Gs proteins and increase the activity of adenylyl cyclase,^49^ leads to a proconvulsive effect^50^ and therefore may reduce the LFS antiepileptogenic action.

Applying LFS following kindling stimulations barricaded the changes in paired‐pulse indices at different inter‐pulse intervals. This action was blocked by sulpiride at a dose that had no significant effect on paired‐pulse indices of kindled animals. Because the variations in paired‐pulse indices are good signs of variations in GABAergic feed‐back and feed‐forward activity of local circuits,^51^, ^52^ another probable mechanism of LFS action may be through changes in GABAergic activity. In fact, the enhancement in the early and late paired‐pulse depression during kindling reflects the increment of GABAergic activity during kindling that may involve in neuronal synchronization during epileptogenesis.^53^ The inhibitory effect of LFS on these parameters may show another antiepileptogenic mechanism of LFS through GABAergic transmission – a mechanism that depends on dopamine D_2_‐like receptors' activity. Our previous experiments showed that LFS prevented the kindling‐induced increase in both glutamatergic and GABAergic currents. These effects were restored when haloperidol (another antagonist of D2‐like receptors) was used before LFS. Therefore, it may be suggested that similar changes occur in the present study by sulpiride.^17^

Another finding of the present study was the D_2_‐like receptors' involvement in inhibitory action of LFS on generation of generalized seizure behavior. This is because the dADD diminished in KLFS group animals and these animals showed only seizure stages 1–3 which are classified as partial seizures.^54^ Obtained results were also in line with our previous hypothesis that the neuromodulators acting through Gi protein signaling may exert role in the anticonvulsant action of LFS. On the other hand, the LFS contributes to the increase in the activity of Gi‐coupled receptors.^12^ Therefore, restoring the inhibitory action of LFS by sulpiride may confirm the role of D_2_‐like receptors in mediating the antiepileptogenic action of LFS.

Sulpiride shows high affinity (at the range of nM) to D_2_ and D_3_ receptors and a lower affinity (at the range of μM) to D_4_ receptors^55^ and it does not block D_1_ dopaminergic, adrenergic, cholinergic, GABAergic, histaminergic, or serotonergic receptors to an appreciable extent.^56^ However, some researchers showed that sulpiride at high doses may act through other neurotransmission systems. Sulpiride has poor affinity for α_1_ ^57^ and α_2_ receptors.^58^ In addition, the effect of 5‐HT_1A_ receptor activation potentiates following D_2_ receptor blockade by sulpiride .^59^ It has also been reported that sulpiride may inhibit the carbonic anhydrase activity.^60^ Therefore, considering the above‐mentioned reports, it seems difficult to rule out that the observed effects of sulpiride in the present study may be because of sulpiride interactions with other receptors rather than purely dopaminergic. In fact, our recent studies suggested a role for adrenergic^61^ and serotonergic receptors.^6^ In addition, inhibition of carbonic anhydrase leads to an anticonvulsant effect.^62^ However, based on the dosage used in this study, the sulpiride action is dominated mainly through D_2_‐like (i.e., D_2_, D_3_ and D_4_) dopamine receptors.

On the whole, it may be concluded that activation of D_2_‐like receptors is crucial for the antiepileptogenic effect of LFS. Considering the fact that applying LFS directly to the seizure focus had anti‐seizure effect in humans^63^ and also decreased the inter‐ictal spikes in temporal lobe epilepsy patients,^64^ it is necessary to find the precise mechanisms involved in the anticonvulsant and antiepileptogenic action of LFS.

## CONFLICT OF INTEREST

The authors declare no conflict of interest.

## Data Availability

The datasets generated during and/or analyzed during the current study are available from the corresponding author on reasonable request.
